# Long-term variation of satellite-based PM2.5 and influence factors over East China

**DOI:** 10.1038/s41598-018-29366-x

**Published:** 2018-08-06

**Authors:** Qianshan He, Fuhai Geng, Chengcai Li, Haizhen Mu, Guangqiang Zhou, Xiaobo Liu, Wei Gao, Yanyu Wang, Tiantao Cheng

**Affiliations:** 1 Shanghai Meteorological Service, Shanghai, 200030 China; 2 Shanghai Key Laboratory of Meteorology and Health, Shanghai, 200030 China; 3 Department of Atmospheric and Oceanic Sciences, School of Physics, Peking University, Beijing, 100871 China; 4 Department of Environmental Science & Engineering, Fudan University, Shanghai, 200438 China

## Abstract

With the explosive economic development of China over the past few decades, air pollution has attracted increasing global concern. Using satellite-based PM_2.5_ data from 2000 to 2015, we found that the available emissions of atmospheric compositions show similar yearly variation trends to PM_2.5_, even if the synchronization is not met for each composition, implying that the intensity of anthropogenic emissions dominates the temporal variation of PM_2.5_ in East China. Empirical orthogonal function analysis demonstrates that the dominant variability in the seasonal PM_2.5_ is closely associated with climate circulation transformation, incarnated as the specific climate index such as the Asia Polar Vortex intensity in spring, the Northern Hemisphere Subtropical High Ridge Position for the leading mode and the Kuroshio Current SST for the second mode in summer, the Asia Polar Vortex Area for the leading mode and the Pacific Polar Vortex Intensity for the second mode in autumn, the NINO A SSTA for the leading mode and the Pacific Decadal Oscillation for the second mode in winter. Therefore, apart from anthropogenic emissions effects, our results also provide robust evidence that over the past 16 years the climate factor has played a significant role in modulating PM_2.5_ in eastern China.

## Introduction

In recent years, pollution (haze) has been very serious, especially in developing countries, which is closely related to the explosive growth of aerosol emissions induced by rapid economic development and urbanization, and is also caused by climate warming due to human activities. East China has experienced rapid industrialization in the last twenty years, which has led to a huge increase of vehicles and industrial emissions. Under the background of global warming, the climate in eastern China has undergone a notable change. Much research has focused on visual range variation in this region^1–3^, and has demonstrated that the obviously increasing haze days per year result in a decreasing trend of visibility over most of the East China region^4^.

Exactly why the haze days per year have grown so vigorously over these years is not clear^5^. The deterioration of air quality is closely related to human activities, including traffic and industrial emissions of pollutants associated with enhanced urbanization and industrialization^6–9^, and natural dust emissions^10,11^, but is not completely an emission phenomenon, special topography, elevation and thermal conditions can significantly impact the aerosol emissions, transport, accumulation and deposition^12^, and the specific weather background has also been favorable for the formation of heavy pollution. East China is located on a monsoon climate influence district, where the meteorological elements, including temperature, precipitation, and atmospheric circulation, are dominated by the obvious seasonal characterization and have acted as the key district for global climate change research. As a result, the generation, emission and transportation process of aerosol will be impacted by the monsoon atmospheric circulation, and even the remote sea surface temperature (SST) in Atlantic Ocean^13^, and become one of the hot research topics in atmospheric environment and climate change^14^. Zhang *et al*.^15^ analyzed the persistent severe fog and haze events over eastern China in January 2013, and concluded that the subdued East Asia winter monsoon led to a shrinking westerly jet, resulting in diminished horizontal wind shear and vertical mixture, which turned into increasing atmospheric stratification stability for more aerosol accumulation. Guo *et al*.^16^ found that wind speed and direction are the critical factors which influence the periodical change of local fog-haze events. Li *et al*.^17^ pointed out that the interannual variation of the wintertime fog-haze days across central and eastern China are dominated by the East Asian winter monsoon. Zhao *et al*.^18^ demonstrated notable decadal fluctuations in the number of haze days during winter in central eastern China, associated with the Pacific Decadal Oscillation (PDO), using a comprehensive observational haze dataset. Numerical model research also indicated that the intensity of the East Asia summer monsoon contributes significantly to the variation of aerosol concentration by wind field, precipitation, and transportation, of organic carbon from biomass burning events^19–22^. It should be noted that the distributions of aerosol are strongly influenced by distinct weather and monsoon circulation, but in turn, it can also be modulated by aerosol effects via reducing surface insolation, absorbing solar radiation and even changing the cloud properties and precipitation, etc^23,24^.

Of all the components of air pollution, particulate matter with an aerodynamic diameter ≤ 2.5 μm (PM_2.5_) is the most significant. Subject to the observation means and model development, knowledge of the interaction between air pollution (aerosol concentration) and the Asia monsoon also remains elusive, especially for the impact of weather conditions, including monsoon circulation and precipitation, on aerosol concentration. Additionally, the yearly-scale fluctuations of PM_2.5_ in East China have not been documented extensively.

The purpose of this paper is to use MODIS (Moderate Resolution Imaging Spectroradiometer) retrieved mass concentration of fine particles to analyze the relationships of these particles with climate indices.

## Data and Methodology

### Data

The Aerosol Optical Depth (AOD) data from MODIS are widely used to investigate the spatial distribution of tropospheric aerosols^25^. The AOD data (Collection 5 before Oct 1, 2014, and after for Collection 6) of MODIS Level 2 products over land (10-km resolution at the nadir) from the Terra satellite calculated by Dark Target algorithm were used to calculate the surface-level aerosol extinction coefficient from February 25, 2000 to December 31, 2015 in this study. The surface-level relative humidity (RH) data from the more than 400 stations used for hygroscopic growth correction in this study were obtained from the China Meteorological Administration (CMA) operation issuing database. The vertical distribution of meteorological parameters (temperature, relative humidity, wind speed, and direction) in each grid was derived from the National Centers for Environmental Prediction final version re-analysis (NCEP FNL) data for calculating the aerosol layer height (ALH) in the vertical correction algorithm. The influence of circulation background on PM_2.5_ variation is also analyzed by using the NCEP FNL data.

Monthly mean NOx surface emission estimates at 0.25 × 0.25 degree resolution, available for the period from 2007 to 2014, are derived from the Ozone Monitoring Instrument (OMI) observations of tropospheric NO2 columns by the DECSO algorithm (Daily Emission estimation Constrained by Satellite Observations, Version 4). An extensive algorithm description can be found in Mijling and Van der A^26^, and the GlobEmission Algorithm Theoretical Baseline Document. Yearly averaged SO2 surface emission estimates at 0.25 × 0.25 degree resolution (version 1.0) are derived from OMI column observations of SO2 columns from 2005 to 2014. Anthropogenic VOC emissions over China for June-July-August 2007–2012 are available at 0.25 × 0.25 degree resolution, which are derived from source inversion, using the adjoin of the IMAGESv2 global chemistry-transport model^27,28^ and constrained by tropospheric HCHO column densities from the OMI satellite instrument^29^. OMI-based CO emissions from agricultural waste burning in Tg m^−1^ or Tg y^−1^ over the North China plain (32–40°N,112.5–120°E) are available at 0.25 × 0.25 degree resolution. All the emission data are provided by the Royal Netherlands Meteorological Institute (KNMI).

A total of 130 monthly climate indices (88 atmospheric circulation indices, 26 ocean temperature indices and 16 other collected indices), provided by National Climate Center of China over the period of 2000–2015 (http://cmdp.ncc.cma.gov.cn/Monitoring/cn_index_130.php), as reference factors associated with PM_2.5_ variability.

### Methodology

We used the vertical correction algorithm of He *et al*.^30^ to obtain the surface-level aerosol extinction coefficient from the MODIS/AOD product, and then applied the algorithm of He *et al*.^31^ to calculate the surface-level PM_2.5_ in a spatial resolution of 10 km × 10 km from aerosol extinction coefficients in East China. Here this will be only briefly described. The key two parameters of retrieving the surface-level aerosol extinction coefficient from AOD are the Planetary Boundary Layer (PBL) height and ALH based on the vertical distribution model, which assumes an exponential aerosol extinction coefficient decay with altitude above the top of PBL. The PBL heights were simulated using the WRF Model (version 3.2.1). A three-domain, two-way nested simulation was implemented in this study. An automated workflow algorithm is applied to calculate the ALH in the whole region, using the vertical distribution of meteorological parameters (temperature, relative humidity, wind speed, and direction) in each grid from NCEP FNL data. The season-long spatial comparison between satellite estimations and the surface-level measurements demonstrates that most of the correlation coefficients (90%) are greater than 0.6, and more than half of the samples (68%) have coefficients higher than 0.7 for the whole data set^30^. An observation-based spatial distribution of indicators describing the integrated humidity effect in East China was introduced to calculated PM_2.5_ mass concentrations with *in situ* RH measurements. Validation of the hygroscopic growth parameters shows good agreement of calculated PM_2.5_ mass concentrations with *in situ* measurements with correlative coefficients of over 0.85^31^.

## Results and Discussions

### Impact of emission on PM_2.5_ distribution variation

**Figure 1:**
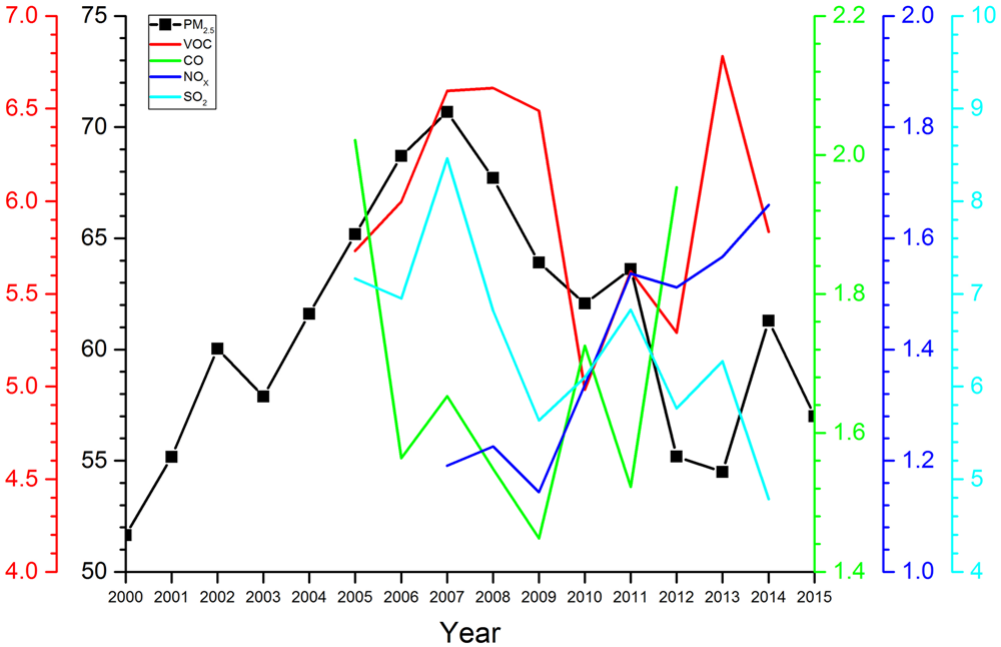
The temporal variation of the regional averaged PM_2.5_ in East China from 2000 to 2015. The available emissions of distinct species are also overlapped against year.

Human activities are the key factor impacting the PM_2.5_ distribution variation. This was inseparable from the rapid development of the Chinese economy, along with an exploding population and increasing pollutant emissions. Figure 1 presents the temporal variation of the regional averaged PM_2.5_ in East China from 2000 to 2015. It was found that the regional averaged PM_2.5_ concentration shows an approximative peak pattern over the last 16 years, with the highest, 70.69 μgm^−3^, and the lowest, 51.65 μgm^−3^, occurring in 2007 and 2000, respectively. The available emissions of distinct species in each year are also overlapped in this figure. It is obvious that the emissions of SO_2_ (Gg y^−1^) show similar yearly variation trend to PM_2.5_, with highest values occurring in 2007 when pollution controls were not a high priority during growth, and primary particles and precursors were emitted into the atmosphere. After that, PM_2.5_ decreases yearly, revealing the effect of energy savings and emissions reduction. After 2010, the emissions of this species fluctuated with the similar trend of PM_2.5_, consistent with the under-controlled pollutant emissions that follow economic growth. Despite the VOC emission data is only available in summer, it could also reflect the variation trend of VOC from year to year due to the relatively stable monthly variation characteristics in East China. The temporal variation of VOC (kg y^−1^) emissions is characterized by two peaks in 2008 and 2013, respectively. In general, the trend of VOC is like that of PM_2.5_ from 2005 to 2014, but for 2013 when VOC emissions increased significantly to the maximum whereas PM_2.5_ decreased to the minimum since 2001. CO emission (kg y^−1^) from agricultural waste burning decreased dramatically from 2005 to 2009, which is approximately identical to the variation of PM_2.5_ from 2007 to 2009. Unlike the above three species, regional average NOx emission increased stably, up to the maximum of 1.66 Gg N y^−1^ in 2014. These atmospheric compositions are all the precursor of aerosol particles, which contribute to form the secondary aerosol by the gas-to-particle chemical process. Therefore, it can be concluded that the intensity of anthropogenic emissions dominates the temporal variation of PM_2.5_ in East China.

### Impact of weather background on PM_2.5_ distribution variation

**Figure 2:**
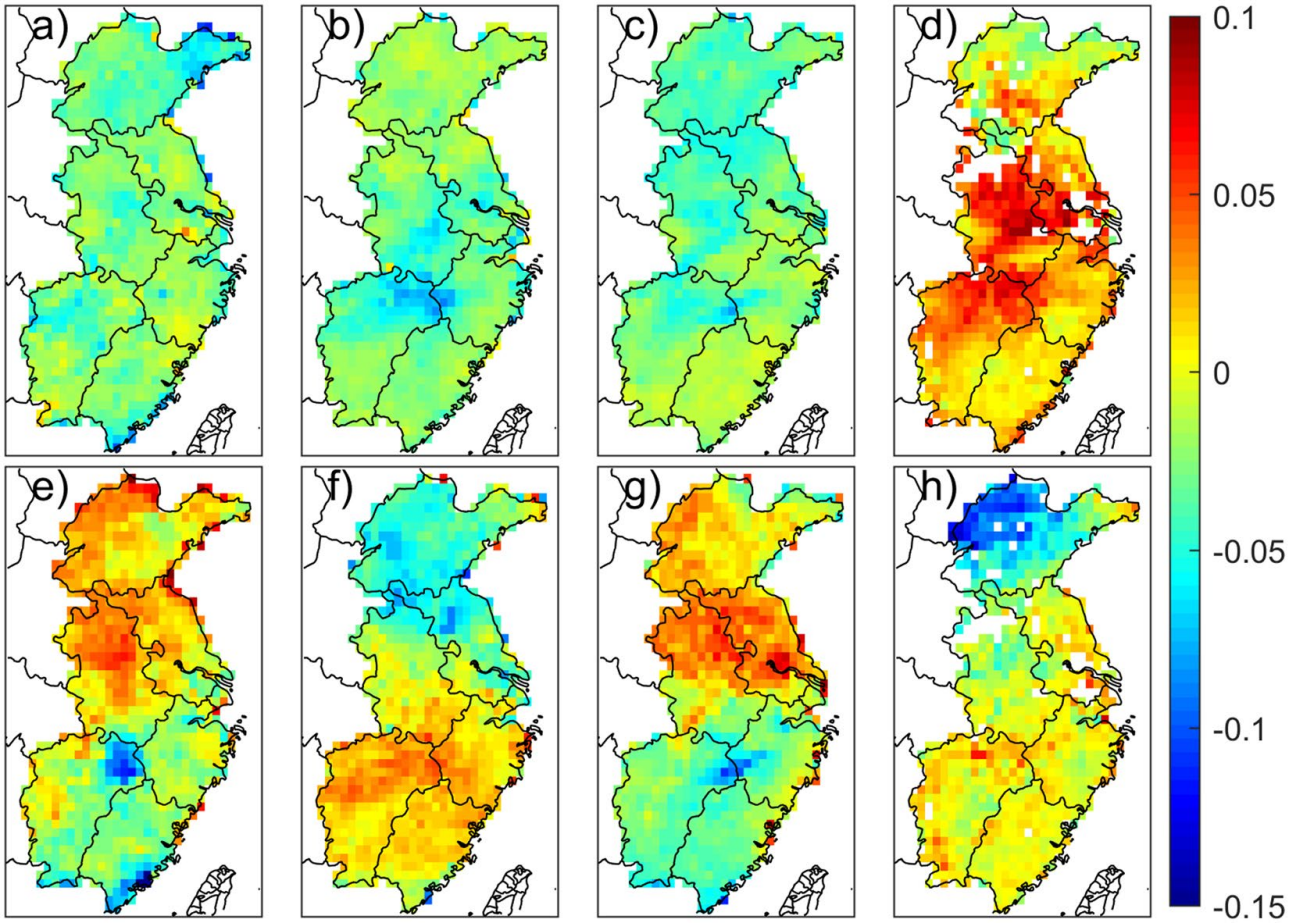
The spatial pattern of the first EOF modes, PC1, in **(a)** Spring, **(b)** Summer, **c)** Autumn, and **(d)** Winter. The second EOF modes, PC2, in **e)** Spring, **(f)** Summer, **(g)** Autumn, and **(h)** Winter. All plots were generated using MATLAB, version 9.0.0.341360, https://cn.mathworks.com/support/?s_tid=gn_supp).

It is apparent that meteorological conditions can impact the generation, distribution, maintenance, and dispersion of local aerosol. Therefore, the transformation of large scale circulation can also affect the transportation and resident time of aerosol in the whole region. We analyzed the impact of weather background on PM_2.5_ distribution variation for each season, due to obviously distinct characteristics of circulation for different seasons in East China. The dominant variability in the seasonal PM_2.5_ was extracted using an empirical orthogonal function (EOF) analysis over eastern China. To lessen the interference from the small-scale local PM_2.5_ fluctuation, a 30 km × 30 km spatial average is used in the seasonal average PM_2.5_ distribution for each year. The contributions of the associated principal component (PC) in the total variance are listed in Table 1, which accounts for at least 38.7% in Spring and a significant 65.9% in Summer of the total variance, and features an almost identical long-term trend in the PM_2.5_ pattern over eastern China. Figure 2 shows the spatial pattern of the first two PCs in each season. These changes to PM_2.5_ are further illustrated by comparing linear trends for PC with climate index in the different seasons.

**Table 1:** The contribution of the first two PCs to the total variance for the four seasons.

Season	Variance contribution proportion (%)	
	PC1	PC2
Spring	24.9	13.8
Summer	50.0	15.9
Autumn	48.4	11.8
Winter	33.9	13.8

### Spring

**Figure 3:**
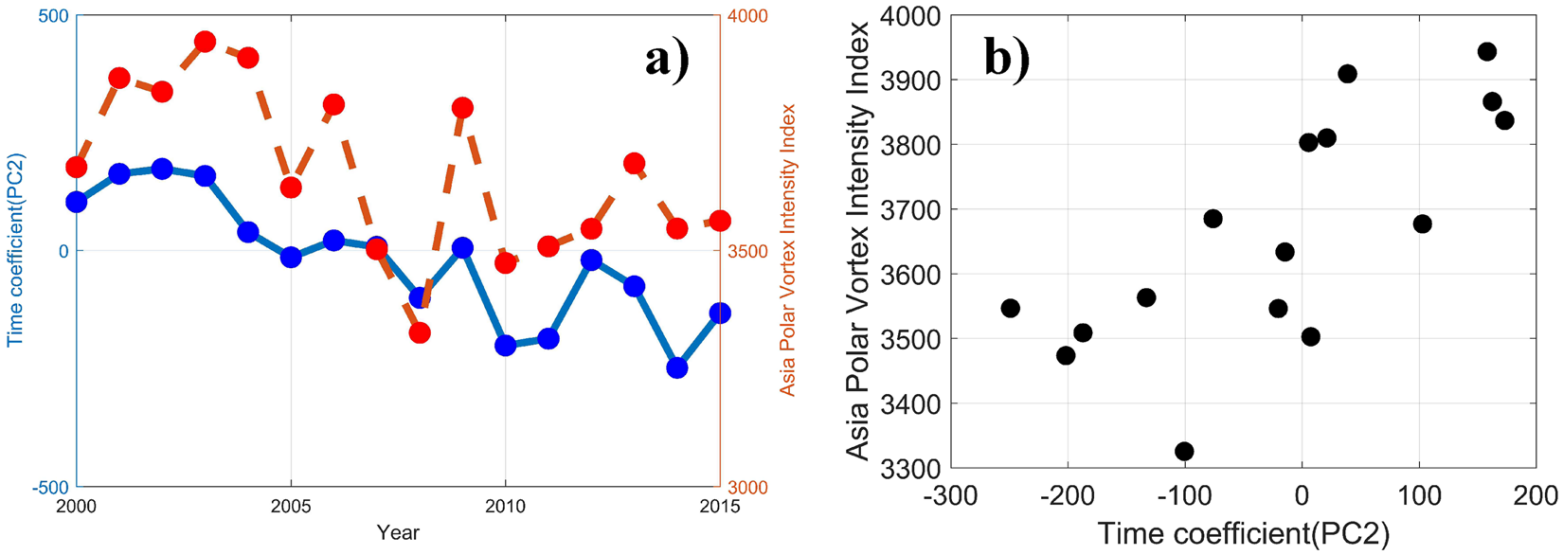
**(a)** The time series and **(b)** the scatter points comparison of PC2 and the APVI. The blue solid line is PC2 with the value and parameter name labeled on the left y-axis and the red dashed line the APVI on the right y-axis. The same as those figures with only two variables hereinafter.

It was found that there is not a significant climate index in our dataset closely connected with the first principal component (PC1) in spring, despite a high variance contribution proportion. The second principal component (PC2, Fig. 2e), which accounts for 13.8% of the total variance, features positive anomalies in the northern part of East China and weaker negative anomalies over the southern part of East China, with large negative anomalies in the northeastern part of Jiangxi province and the coastal area of Fujian province. The PC2 time series shows a distinct downward trend since 2002, and a yearly variation that closely follows the spring Asia Polar Vortex Intensity Index (APVI, see definition in S1) with a correlation coefficient of 0.75 (Fig. 3), indicating that the spring PM_2.5_ in eastern China is possibly related to the APVI.

The polar vortex is one of the important systems of atmospheric circulation in the high latitude region, which acts as a cold vortex near the pole. Its intensity and location are closely related to the weather changes and cold air activities in China. Strong polar vortex activity can lead to spring chilling weather in southern China^32^. As shown in Fig. S1(b), the center of negative anomaly of the 500 hPa geopotential height field is located in the Eurasia continent in the year of 2003, with the most intense Polar vortex, which leads to an enhancement of the East Asian Trough, resulting in more pollutants transported to East China from North China by cold air mass.

#### Summer

**Figure 4:**
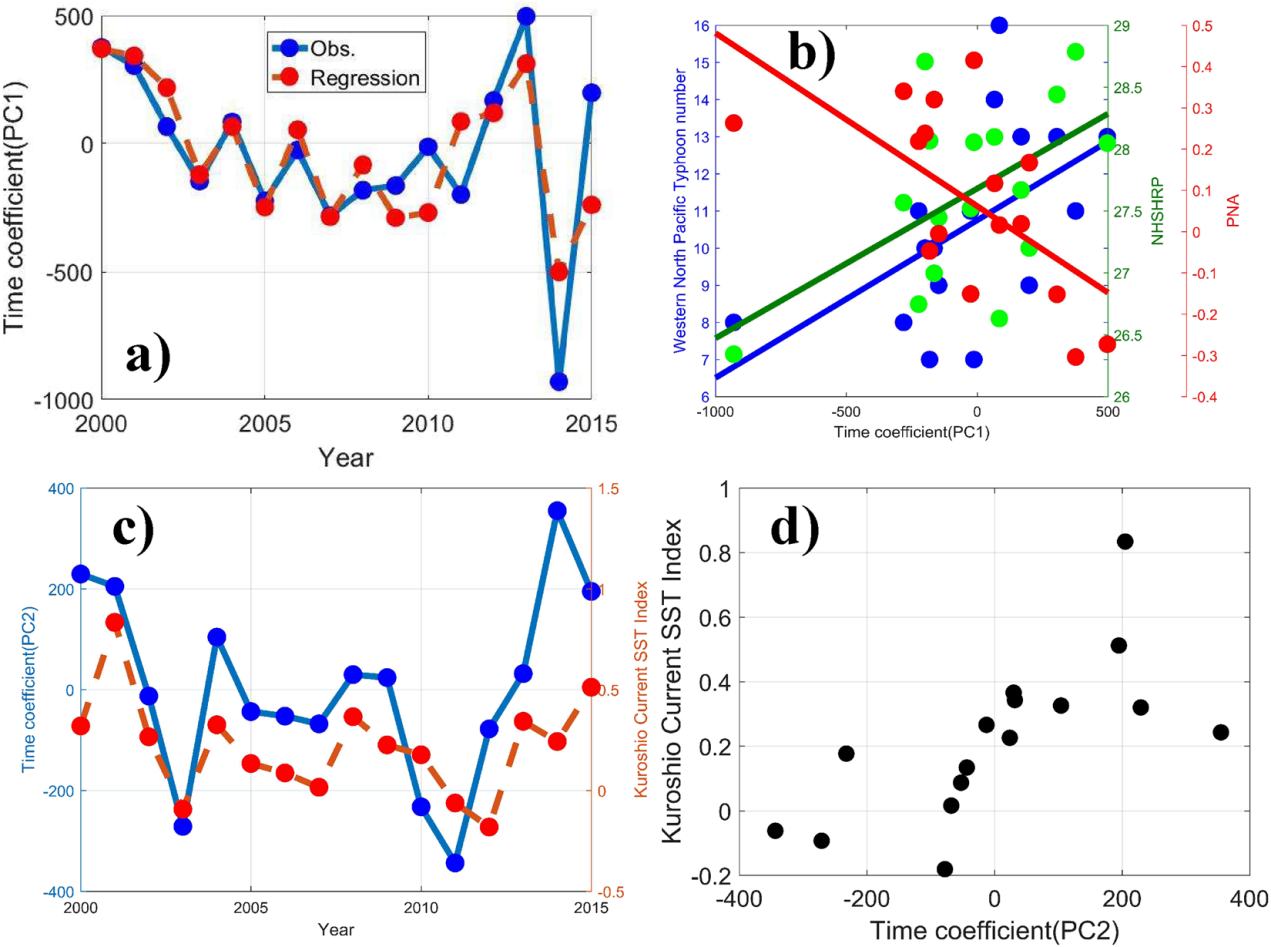
**(a)** The time series of PC1 and the regression line from the Western North Pacific Typhoon number, the NHSHRP, and the PNA. **(b)** The scatter points comparison and fitted lines of PC1 with the different indices. **(c)** The time series and **(d)** the scatter points comparison of PC2 and the KCSSTI.

The PC1 spatial pattern is characterized by positive anomalies in the most northern and southern parts of East China and weaker negative anomalies over the Boyang Lake plain and Huai River plain, with large negative anomalies in the border between Jiangxi province and Zhejiang province (Fig. 2b). The associated PC1 time series shows a marked decreasing trend before 2007, and remains week shaking variation until 2011 with a switch from the positive phase (2012–2013) to the negative phase (2014), which is in phase with three indexes of the Western North Pacific Typhoon number (R = 0.54), the Northern Hemisphere Subtropical High Ridge Position Index (NHSHRP, R = 0.55), and the Pacific/North American Pattern (PNA, R = −0.63) (see definition in S2), as shown in Fig. 4(a,b).

The close relation between PM_2.5_ and Western North Pacific Typhoon number is possibly induced by the ocean temperature anomalies (SSTA), and the typhoon number is an indicator of SSTA. Wu and Chen^33^ found that an oscillation period of PNA of 3 to 5 years is coincident with that of the ENSO.

A multivariate regression is adopted to obtain the weight factor of the three indices for their contribution to PC1 variation. The time coefficient of PC1 (*T*) can be calculated as

1 $$T=68.8{x}_{1}+247.0{x}_{2}-0.2{x}_{3}-7588.8$$

Where *x*_1_, *x*_2_, and *x*_3_ are the Western North Pacific Typhoon number, NHSHRP, and PNA, respectively.

The regression line of *T* shows notable consistency with the time coefficient of PC1, indicating that the summer PM_2.5_ in eastern China is closely related to the combined action of the three indices. To understand the underlying mechanism of the relationship between the three indices and PM_2.5_ yearly mode, we found that the PM_2.5_ yearly mode is significantly correlated with the 850 hPa vertical velocity field over eastern China, especially for central eastern China, with positive correlation and others with negative correlation (Fig. S2), indicating that the dynamical stability may play a key role in determining the PM_2.5_ yearly variations. During the positive phase of the time series of PC1, stronger vertical transportation in central eastern China is conducive to aerosol diffusion and results in less PM_2.5_ over central eastern China, while the weaker vertical transportation over northern and southeastern China prevents pollutants in the PBL from being mixed upward into the free troposphere, and leads to more PM_2.5_ in this region.

The summer PC2 spatial distribution pattern shows positive anomalies in the southern part of East China, with maximum positive anomalies in the Boyang Lake plain, and negative anomalies in the northern part of East China, with minimum negative anomalies in the border among Anhui, Jiangsu, and Shandong provinces (Fig. 2b). The associated PC2 time series has correlation coefficients of 0.68, with the summer Kuroshio Current SST Index (KCSST, see definition in S3) (Fig. 4c,d). Kuroshio is a powerful warm current in the northwestern Pacific, where the net heat release is the biggest in the global oceans, resulting in a large amount of energy released in this area transported to the northern hemisphere^34^. The sea surface thermodynamic conditions in this area are closely related to the climate and weather in China. Early in the 1970s, research showed that the summer precipitation in the middle and lower reaches of the Yangtze river had obvious positive correlation with the SST in the Kuroshio area^35–37^.

To gain better insight into the potential influence of the KCSST on yearly changes in PM_2.5_, Figure S3 presents the correlation between annual summer mean surface horizontal wind velocity in each grid over eastern China and the PC2 for the period of 2000–2015. The significantly positive correlation of PC2 with the surface horizontal wind velocity field over northern eastern China and negative correlation over southeastern China suggests that the sea surface temperature anomaly causes the difference in surface horizontal wind velocity field between northern and southeastern China. The stronger wind over northeastern China during the positive phase of KCSST is favorable to the dispersion of pollutants, consistently resulting in less PM_2.5_ over northeastern China.

#### Autumn

**Figure 5:**
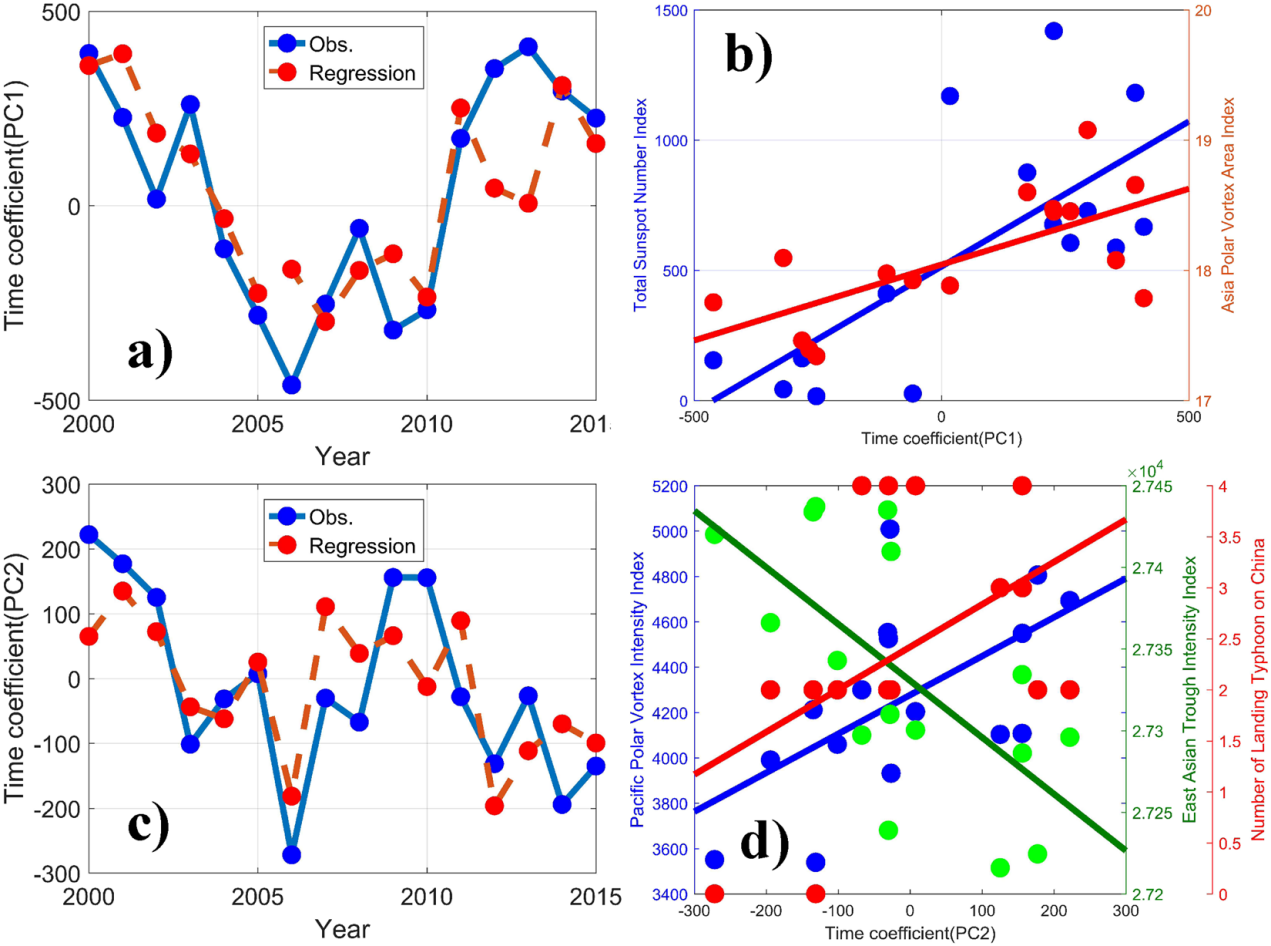
**(a)** The time series of PC1 and the regression line from the TSN and the APVA. **(b)** The scatter points comparison and fitted lines of PC1 with the different indices. **(c)** The time series of PC2 and the regression line from the PPVI, the NLT on China, and the EATI. **(d)** The scatter points comparison and fitted lines of PC2 with the different indices.

The PC1 spatial pattern presents positive anomalies in the Yangtz River Delta region and southern part of East China, except for the border between Jiangxi province and Zhejiang province, and weaker negative anomalies over the northern part of East China (Fig. 2c). As shown in Fig. 5(a,b), the associated PC1 time series exhibits valley-like yearly variations, with the turning point in 2006, strongly in phase with the Total Sunspot Number Index (TSN) and the Asia Polar Vortex Area Index (APVA, see definition in S4), with correlation coefficients of 0.72 and 0.68, respectively.

TSN is a good proxy for the intensity of solar activity that impacts significantly not only the sun-earth space environment, but also the terrestrial atmosphere condition, in particularly the variation of precipitation and temperature^38–41^. The variation of solar ultraviolet radiation can change the ozone content in the stratosphere, further giving rise to the transformation of temperature field and zonal wind in the stratosphere. The atmospheric circulation of the troposphere, influenced by following the B-D circulation and S-T coupling mechanism, play a final role in the variation of PM_2.5_. Previous research found that the Walker circulation associated motion upward over the western equatorial pacific and downward over the eastern pacific become stronger in the year of intense solar activity^42,43^.

A multivariate regression is adopted to obtain the weight factor of the two indices for their contribution to PC1 variation. The time coefficient of PC1 (*T*) can be calculated as

2 $$T=0.32{x}_{1}+219.59{x}_{2}-4110.79$$

Where *x*_1_ and *x*_2_ are TSN and APVA, respectively.

The regression line of *T* shows notable consistency with the time coefficient of PC1 of EOF, indicating that the autumn PM_2.5_ in eastern China is closely related to the combined action of the two indices. Similar to the PC2 of the summer, Figure S4 presents the correlation between annual summer mean surface horizontal wind velocity in each grid over eastern China and the PC1 of autumn for the period of 2000–2015. The significantly positive correlation of PC1 with the surface horizontal wind velocity field over northeastern China and the negative correlation over southeastern China, except for the line of Jiangxi border on Fujian province, is similar to the spatial mode of PC1, which suggests that the variation of the two indices causes the difference in surface horizontal wind velocity field between northern and southeastern China. The stronger wind over northeastern China during the positive phase of the two indices is favorable for the dispersion of pollutants, consistently resulting in less PM_2.5_ over northeastern China and the border of Jiangxi province.

The PC2 spatial pattern shows positive anomalies in the most northern part of East China and weaker negative anomalies in the southern part of East China, with large negative anomalies in the border between Jiangxi province and Zhejiang province (Fig. 2g). The associated PC2 time series shows a distinct fluctuation with a downward trend from 2000 to 2006 and upward until 2009, and then dropping to the minimum in 2014. The variation trend of PC2 is followed closely by the Pacific Polar Vortex Intensity Index (PPVI, R = 0.59) and the Number of Landing Typhoon on China (NLT, R = 0.47), and is correlated reciprocally with the East Asian Trough Intensity Index (EATI, R = −0.65) (see definition in S5), as shown in Fig. 5(c,d). The East Asian trough is an important circulation system in the middle troposphere over East Asia. Its strength and position affect the droughts and floods in autumn and winter in China^44^.

A multivariate regression is adopted to obtain the weight factor of the three indices for their contributions to PC2 variation. The time coefficient of PC2 (*T*) can be calculated as

3 $$T=0.11{x}_{1}-0.80{x}_{2}+9.44{x}_{3}+21362.98$$

Where *x*_1_, *x*_2_, and *x*_3_ are the PPVI, the EATI, and the NLT, respectively.

The regression line of *T* shows notable consistency with the time coefficient of PC2, indicating that the autumn PM_2.5_ in eastern China is closely related to the combined action of the three indices.

The circulation patterns are transformed due to the variation of the above three indices. Figure S5 shows the correlation between PC2 and annual autumn mean 850 hPa geopotential height, sea-level pressure, and surface horizontal wind velocity in each grid for the period of 2000–2015. For the strong East Asia trough and Pacific Polar Vortex, the enhanced deep high-pressure system dominates over Eastern China, characterized by the strong sinking motion abnormalities in the whole layer atmosphere. As a result, the diffusion and transport of pollutants are restrained. Meanwhile, the stronger wind velocity in southeastern China benefits the diffusion of air pollutants when the PC2 is in positive phase.

#### Winter

**Figure 6:**
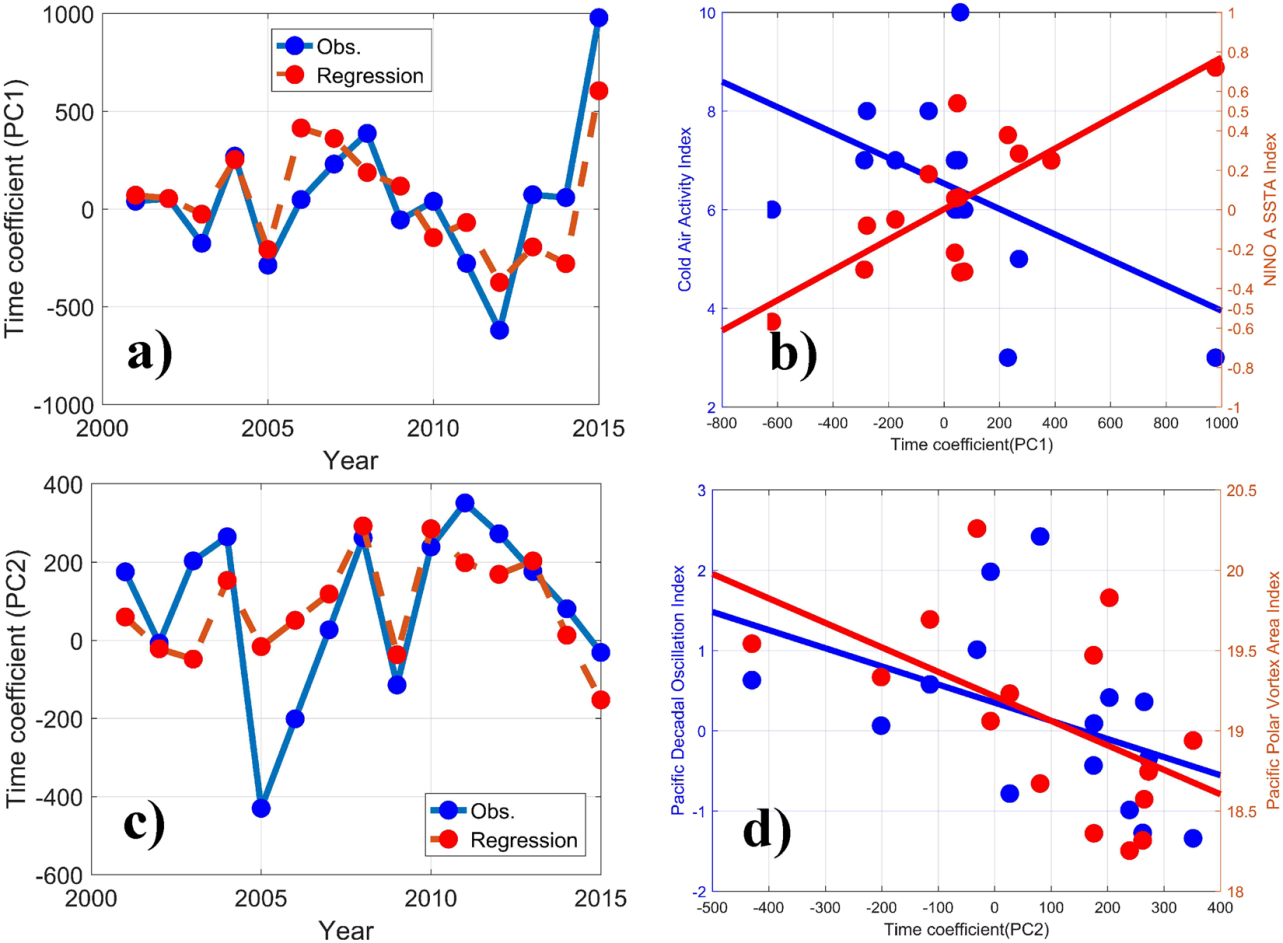
**(a)** The time series of PC1 and the regression line from the CAA and the NINO A SSTA Index. **(b)** The scatter points comparison and fitted lines of PC1 with the different indices. **(**c**)** The time series of PC2 and the regression line from the PDO and the PPVA. **(d)** The scatter points comparison and fitted lines of PC1 with the different indices.

The PC1 spatial distribution pattern of the wintertime PM_2.5_ anomaly shows significant positive anomalies in the central region, weak negative anomalies in the south, and significant negative anomalies in the north of East China (Fig. 2d). The PC1 time series reveals a frequent oscillation with slowing trend before 2008 and then abrupt change, falling to the minimum at 2012. The variation trend of PC1 is correlated reciprocally with the Cold Air Activity Index (CAA, R = −0.51) and followed closely by the NINO A SSTA Index (R = 0.77) (see definition in S6), as shown in Fig. 6(a,b).

A multivariate regression is adopted to obtain the weight factor of the two indices for their contributions to PC1 variation. The time coefficient of PC1 (*T*) can be calculated as

4 $$T=152.99-20.55{x}_{1}+713.14{x}_{2}$$

Where *x*_1_ and *x*_2_ are the CAA and the NINO A SSTA Index, respectively.

The regression line of *T* shows notable consistency with the time coefficient of PC1, indicating that the winter PM_2.5_ in eastern China is closely related to the combined action of the two indices.

The CAA and the NINO A SSTA are the important influence factors on the transformation of circulation patterns in eastern China. As shown in Figure S6, for the positive phase of PC1 in winter, the high pressure is accompanied by a strongly sinking motion in central eastern China. The wintertime mean planetary boundary layer (PBL) height and surface horizontal wind velocity are negatively correlated with the PC1 in central eastern China, resulting in a suppression of vertical and horizontal diffusion of aerosol. The spatial mode of PC1 is perfectly identical with the circulation patterns, indicating the main mechanism of weather background impacting PM_2.5_ variation in eastern China.

The PC2 spatial distribution pattern of the wintertime PM_2.5_ anomaly shows significant positive anomalies in most of East China, except for Shandong province, with outstanding negative anomalies (Fig. 2h). The PC2 time series appeared to have a prominent periodic fluctuation with a 5–6 years interval, achieved nadir at 2005, and peaked at 2011, which is correlated reciprocally with the PDO (R = −0.44) and the Pacific Polar Vortex Area Index (PPVA, R = −0.54) (see definition in S7), as shown in Fig. 6(c,d).

A multivariate regression is adopted to obtain the weight factor of the two indices for their contributions to PC2 variation. The time coefficient of PC2 (*T*) can be calculated as

5 $$T=3110.16-60.56{x}_{1}-158.03{x}_{2}$$

Where *x*_1_ and *x*_2_ are the PDO and the PPVA, respectively.

The regression line of *T* shows notable consistency with the time coefficient of PC2, indicating that the winter PM_2.5_ in eastern China is closely related to the combined action of the two indices. The wintertime climatological circulation over East China in the lower troposphere is dominated by the cold high, on which the activities of the PDO and the PPVA commonly exert their impact. The significant negative correlation of PC2 with the 500 hPa geopotential height in northeastern China (Fig. S7(a)) implies that more cold air activity is favorable for pollutant diffusion during the positive phase of PC2, although a positive correlation of precipitation with PC2 is obvious in both Shangdong (north) and Fujian province (south) (Fig. S7(b)), which implies that less PM_2.5_ is induced by an effective wet removal of aerosol. A remarkable negative correlation of PC2 with PBL height suggests that more pollutants are confined to surface level during the positive phase of PC2. Therefore, the circulation pattern and meteorological conditions determine the different PM_2.5_ concentration in Shangdong province from the other regions in eastern China.

## Conclusion

In this study, the daily surface-level PM_2.5_ with a spatial resolution of 10 km × 10 km in East China, derived from MODIS/AOD by the algorithm of He *et al*.^30,31^ from 2000 to 2015, is used to analyze the influence factors on the temporal-spatial variation of PM_2.5_.

The available emissions of atmospheric compositions show similar yearly variation trends to PM_2.5_, even if the synchronization is not met for each composition, implying that the intensity of anthropogenic emissions dominates the temporal variation of PM_2.5_ in East China.

The dominant variability in the seasonal PM_2.5_ was extracted using an EOF analysis over eastern China, and the changes to PM_2.5_ are further illustrated by comparing with the climate index. The PC2 time series closely follows the spring APVI, with a correlation coefficient of 0.75, indicating that the spring PM_2.5_ in eastern China is possibly related to the Asia Polar Vortex intensity, which transported more pollutants to East China from North China by enhancing the East Asian Trough when the Asia Polar Vortex intensity increased. The summer PC1 time series is in phase with three indices of the Western North Pacific Typhoon number, NHSHRP, and PNA, which influence significantly the vertical velocity field and change the PM_2.5_ distribution. The summer PC2 time series has a high correlation with the summer KCSST, which changes the surface horizontal wind field and further causes the difference of PM_2.5_ between northern and southeastern China. The autumn PC1 presents a strong correlation with the TSN and the APVA, which also impact the PM_2.5_ distribution by changing the surface horizontal wind field. The variation trend of PC2 is followed closely by the PPVI, the NLT on China, and the EATI, which impact the diffusion conditions by changing the pressure system of the whole layer atmosphere and surface-level wind field. The variation trend of the winter PC1 is correlated reciprocally with the CAA and followed closely by the NINO A SSTA Index, while the PC2 is closely connected with the PDO and the PPVA.

Overall, apart from anthropogenic emissions effects, our results also provide robust evidence that in the past 16 years the climate factor has played a significant role in modulating the PM_2.5_ in eastern China. In future work, it will be necessary to introduce the numerical model for distinguishing the relative contributions of anthropogenic emissions and atmospheric conditions.

## Electronic supplementary material

Supplementary Information
