# Supplementary material for: Long-term variation of satellite-based PM2.5 and influence factors over East China
Source: Sci Rep. 2018 Aug 6;8:11764. doi: 10.1038/s41598-018-29366-x (PMC6078948; doi:10.1038/s41598-018-29366-x)
Supplement: Supplementary file 1 — Supplementary Information [file 41598_2018_29366_MOESM1_ESM.pdf]

Supplementary Information for

Long-term variation of satellite-based PM2.5 and influence factors over East China

Qianshan He[1, 2], Fuhai Geng[1]*, Chengcai Li[3], Haizhen Mu[1], Guangqiang Zhou[1, 2], Xiaobo Liu[1], Wei Gao[1], Yanyu Wang[4], Tiantao Cheng[4]

[1]Shanghai Meteorological Service, Shanghai 200030, China. [2]Shanghai Key Laboratory of Meteorology and Health, Shanghai 200030, China. [3]Department of Atmospheric and Oceanic Sciences, School of Physics, Peking University, Beijing 100871, China. [4]Department of Environmental Science & Engineering, Fudan University, Shanghai 200438, China. Correspondence and requests for materials should be addressed to Fuhai Geng (email: fuhaigeng@263.net)

This file includes Supplementary definition S1-S7 and Supplementary Figs. S1–S7, which can be classified as follows:

S1:

The APVI is defined as the total mass of air in the isohypsic surface between 500 hPa isostatic surface and polar vortex south bound characteristic isohypsic surface over the 60°E-150°E of the Northern Hemisphere, and can be calculated as

$$Q = \rho R^2 \Delta\varphi\Delta\lambda \sum_i \sum_j (H_0 - H_{ij}) \cos\varphi_i \qquad (2)$$

Where ρ atmospheric density, R the radius of the earth, and Δφ and Δλ are the difference of latitude and longitude of the adjacent grid on the monthly average geopotential height of 500 hPa, with the grid interval in 10 degrees and 5 degrees, respectively. H_0 is the geopotential height of the polar vortex south bound characteristic isohypsic surface, and H_{ij} is the geopotential height on the northern grids of the polar vortex south bound characteristic isohypsic surface. ρR^2 can be considered as a constant of 0.1.

S2:

The Western North Pacific Typhoon number is the number of tropical cyclones with a maximum surface wind over 8 grade that generate or enter near the center of the Pacific and south China sea, at the north of the equator and west of the date line. NHSHRP represents the average latitude of the location of the subtropical high ridge center on 500 hPa at each meridian in the region of 10°N-60°N and 5°E-360°. The center can be identified by the conditions of zonal wind u=0 and $\partial u / \partial y$ >0. PNA, defined as the time coefficient of PC2 of the 500 hPa geopotential height field in the region of 20°N-90°N and 0-360°, is one of the main circulation structures in the northern hemisphere mid-latitude region, and the most significant modal to indicate the circulation change in the middle and upper troposphere. It describes the atmospheric circulation anomalies in the north Pacific and North America region.

S3:

The KCSST is defined as the regional average sea surface temperature anomaly in the range of 35°N, 140°-150°E and 25°N-30°N, 125°-150°E.

S4:

APVA, defined as the sector area of the 500 hPa geopotential height field in the north region of the polar vortex southern boundary isohypse line between 60°E and 150°E, can be calculated as

$$S = \int_{\varphi}^{\frac{\pi}{2}} \int_{\lambda 1}^{\lambda 2} R^2 \cos\varphi d\varphi d\lambda = R^2 (1 - \sin\varphi)(\lambda_2 - \lambda_1) \qquad (4)$$

Where the unit of S is 10^5 km^2, λ_2 and λ_1 are the longitude in radians, φ the latitude of the polar vortex southern boundary, and R the earth radius (6378 km).

S5:

The PPVI is defined as the total mass of air in the isohypsic surface between the 500 hPa isostatic surface and the polar vortex south bound characteristic isohypsic surface over the 150°E-120°W of the Northern Hemisphere. The EATI can be calculated by the sum minus the maximum and plus the minimum of 500 hPa geopotential height on the trough-line in the range of 30°N-55°N and 110°E-170°E.

S6:

The CAA is the amount of cold air process, which meets a continuous temperature

decreasing more than 5°C in three days at some designated meteorological stations. The NINO A SSTA Index is the regional average of SSTA in the range of 25°N-35°N and 130°E-150°E.

S7:

The PDO is defined as the PC1 time coefficient of SSTA north of 20°N in the Pacific region. The PPVA is defined as the sector area of the 500 hPa geopotential height field in the north region of the polar vortex southern boundary isohypse line between 150°E and 120°W.

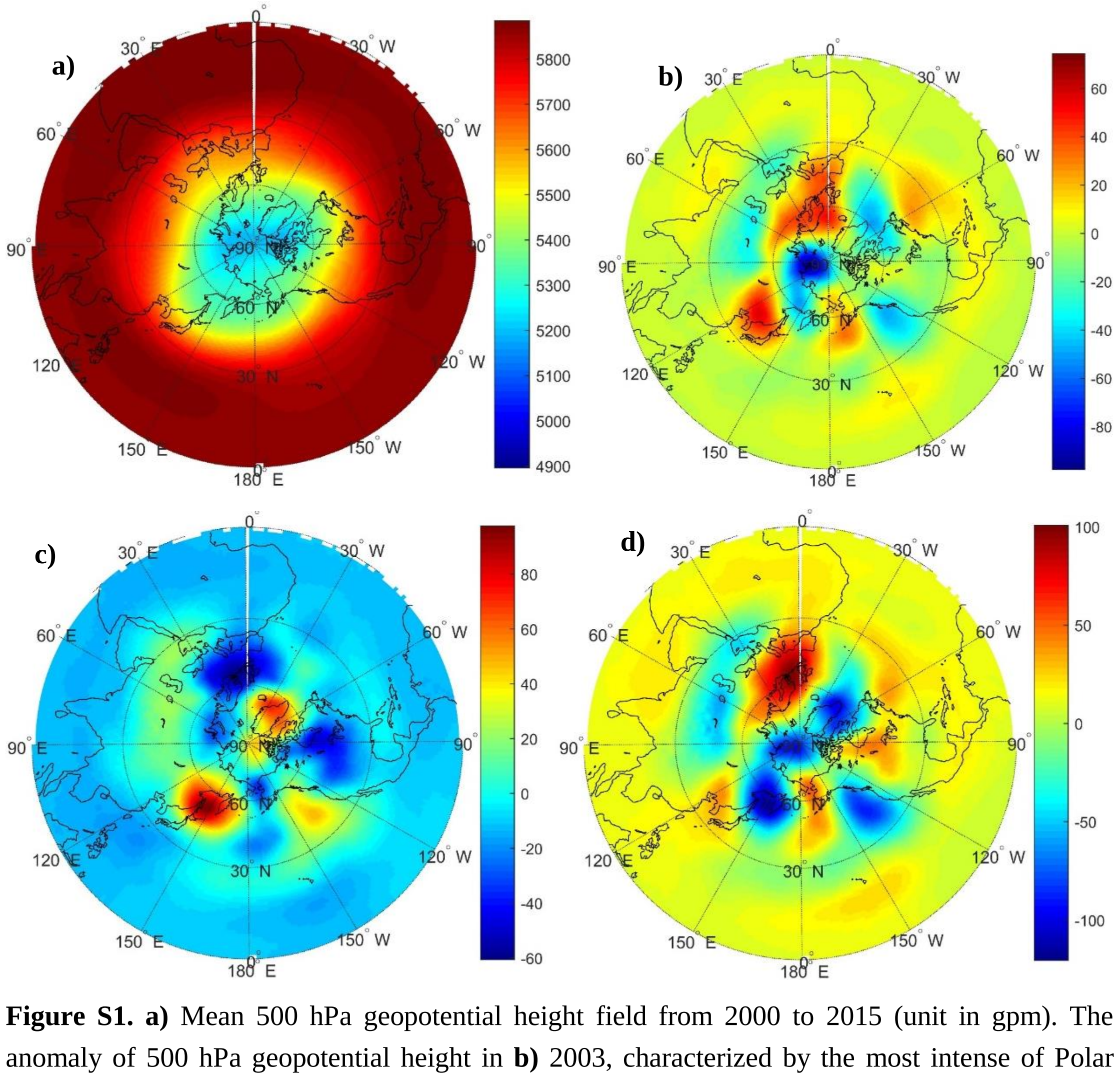

Figure S1. a) Mean 500 hPa geopotential height field from 2000 to 2015 (unit in gpm). The anomaly of 500 hPa geopotential height in **b)** 2003, characterized by the most intense of Polar vortex, and **c)** 2008, by the least intense. **d)** The difference of 500 hPa geopotential height between 2003 and 2008. All plots were generated using MATLAB, version 9.0.0.341360, https://cn.mathworks.com/support/?s_tid=gn_supp).

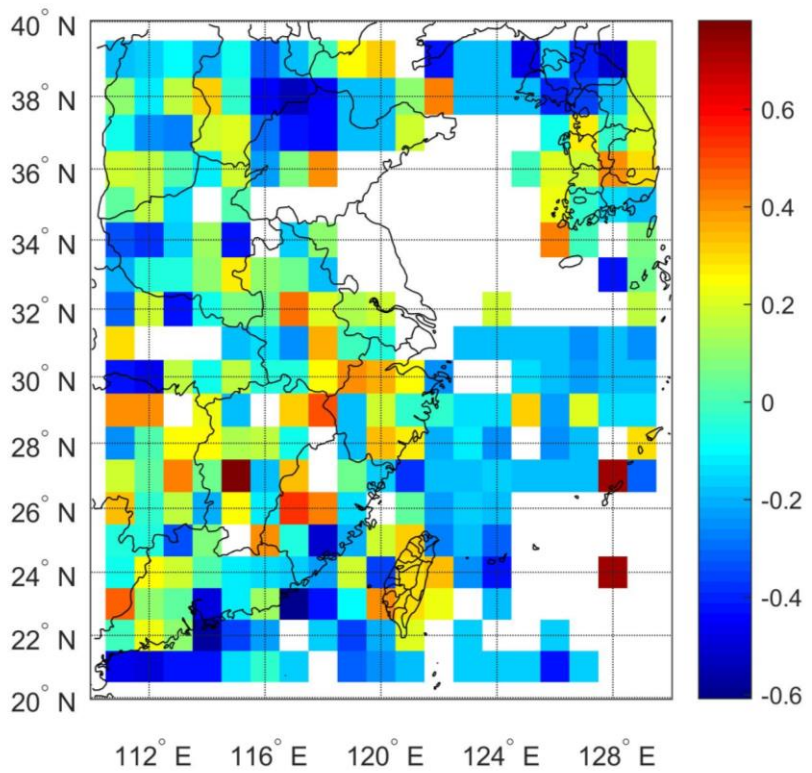

Figure S2. Correlation between annual summer mean vertical velocity on 850hPa in each grid and the PC1 for the period of 2000-2015. This plot was generated using MATLAB, version 9.0.0.341360, https://cn.mathworks.com/support/?s_tid=gn_supp).

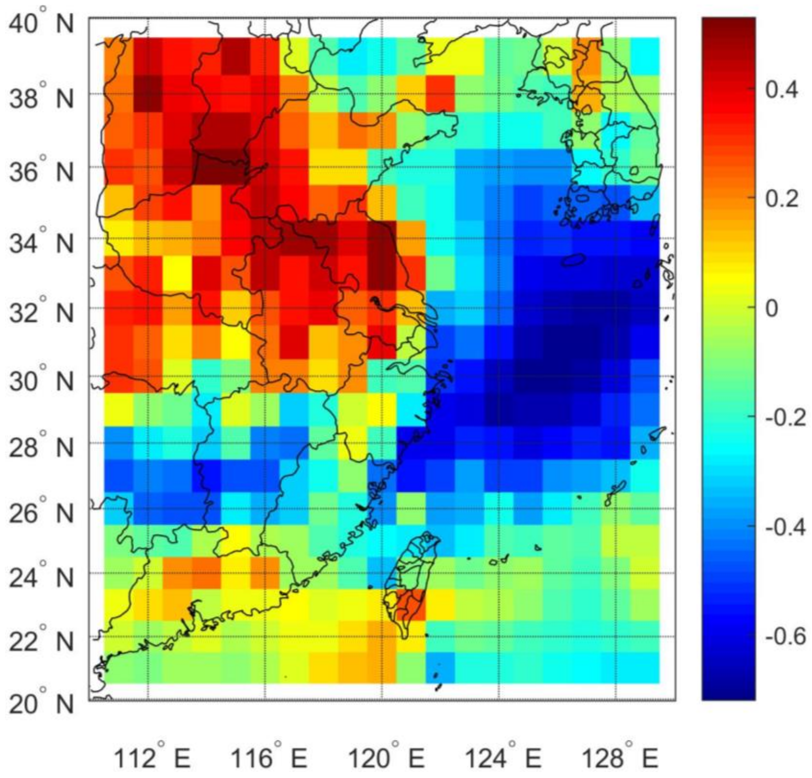

Figure S3. Correlation between annual summer mean surface horizontal wind velocity in each grid and the PC2 for the period of 2000-2015. This plot was generated using MATLAB, version 9.0.0.341360, https://cn.mathworks.com/support/?s_tid=gn_supp).

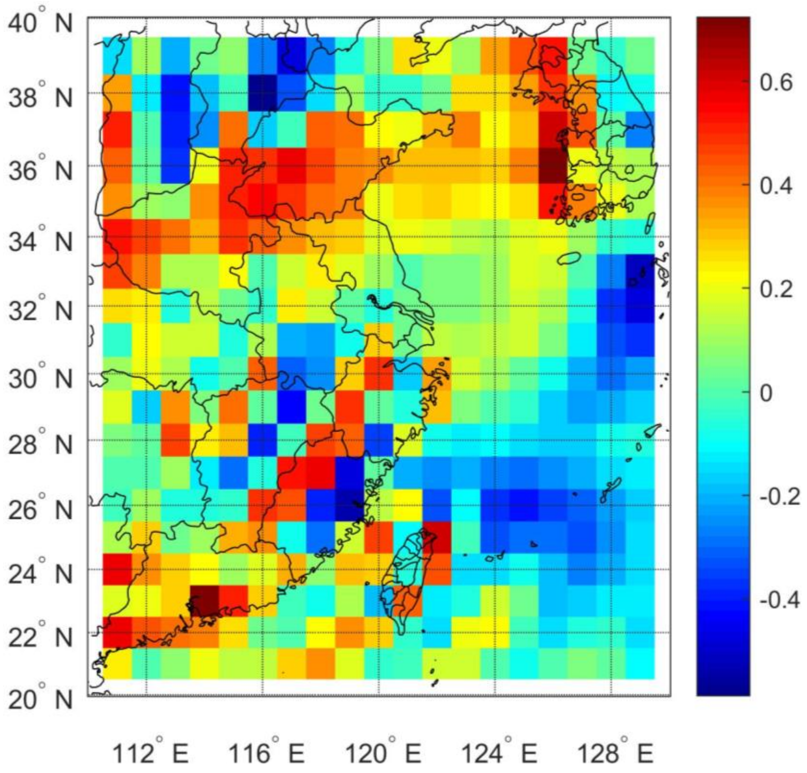

Figure S4. Same as Figure S3, but for PC1 of autumn. This plot was generated using MATLAB, version 9.0.0.341360, https://cn.mathworks.com/support/?s_tid=gn_supp).

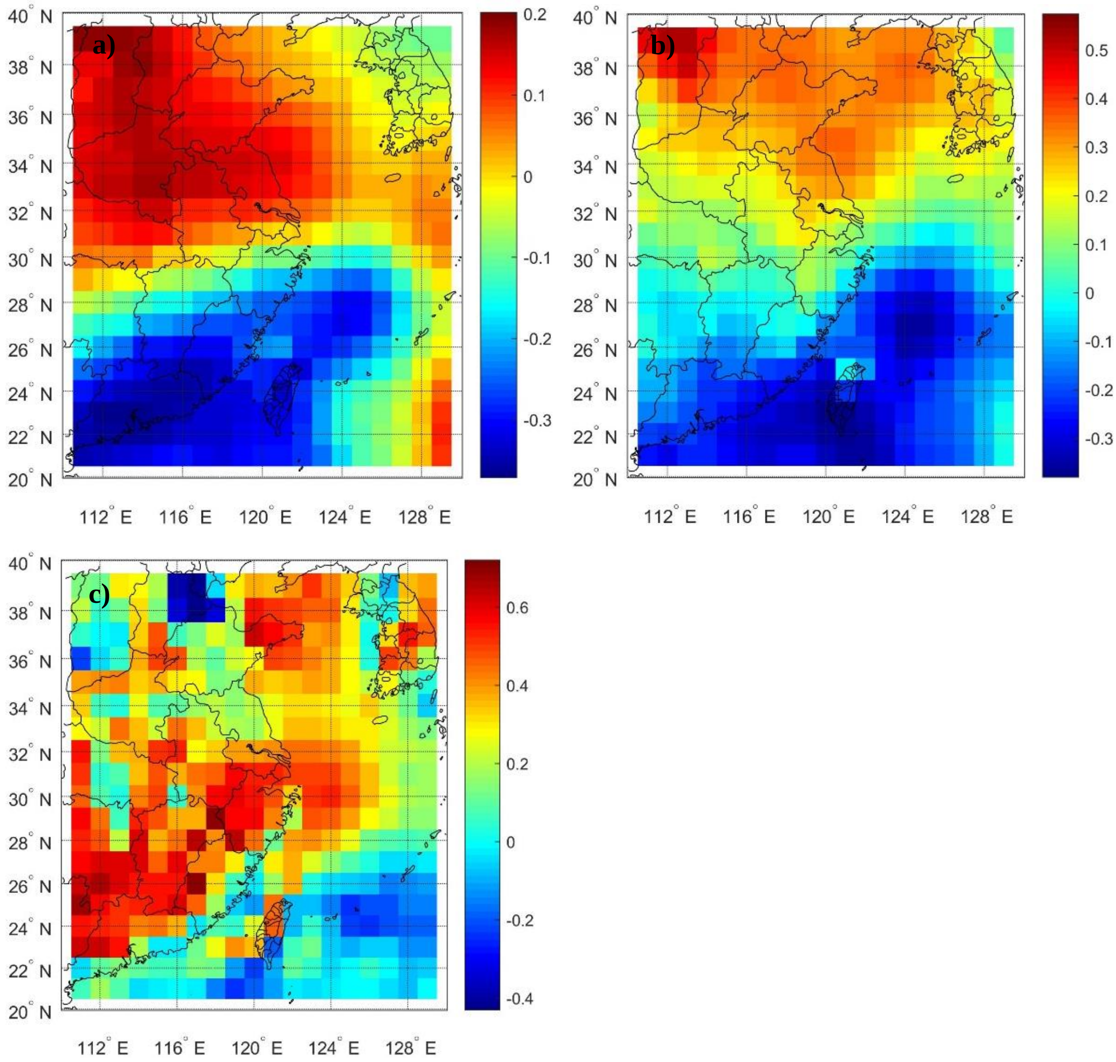

Figure S5. The correlation between PC2 and annual autumn mean **a)** 850 hPa geopotential height, **b)** sea-level pressure, and **c)** surface horizontal wind velocity in each grid for the period of 2000-2015. All plots were generated using MATLAB, version 9.0.0.341360, https://cn.mathworks.com/support/?s_tid=gn_supp).

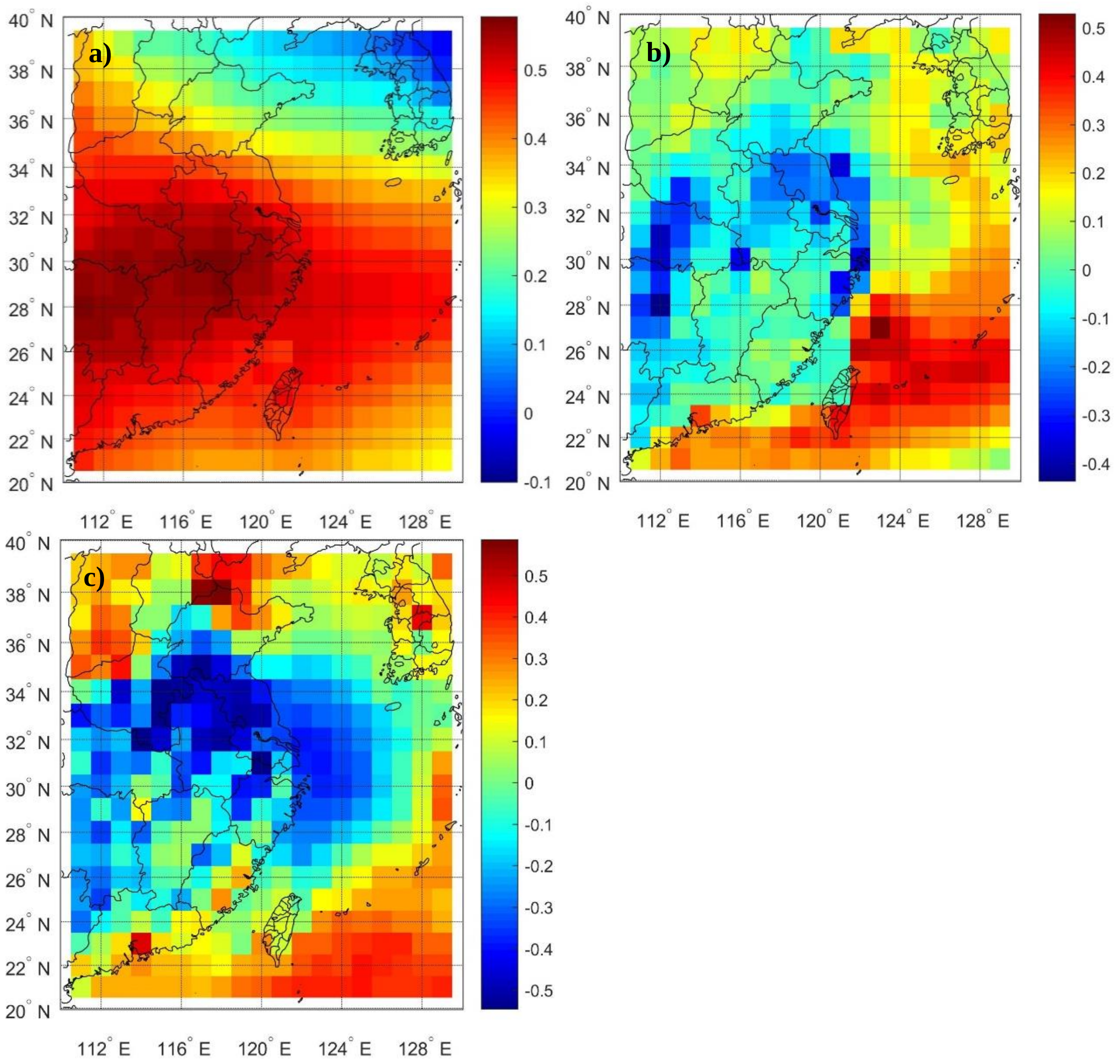

Figure S6. The correlation between PC1 and annual winter mean **a)** 850 hPa geopotential height, **b)** PBL height, and **c)** surface horizontal wind velocity in each grid for the period of 2000-2015. All plots were generated using MATLAB, version 9.0.0.341360, https://cn.mathworks.com/support/?s_tid=gn_supp).

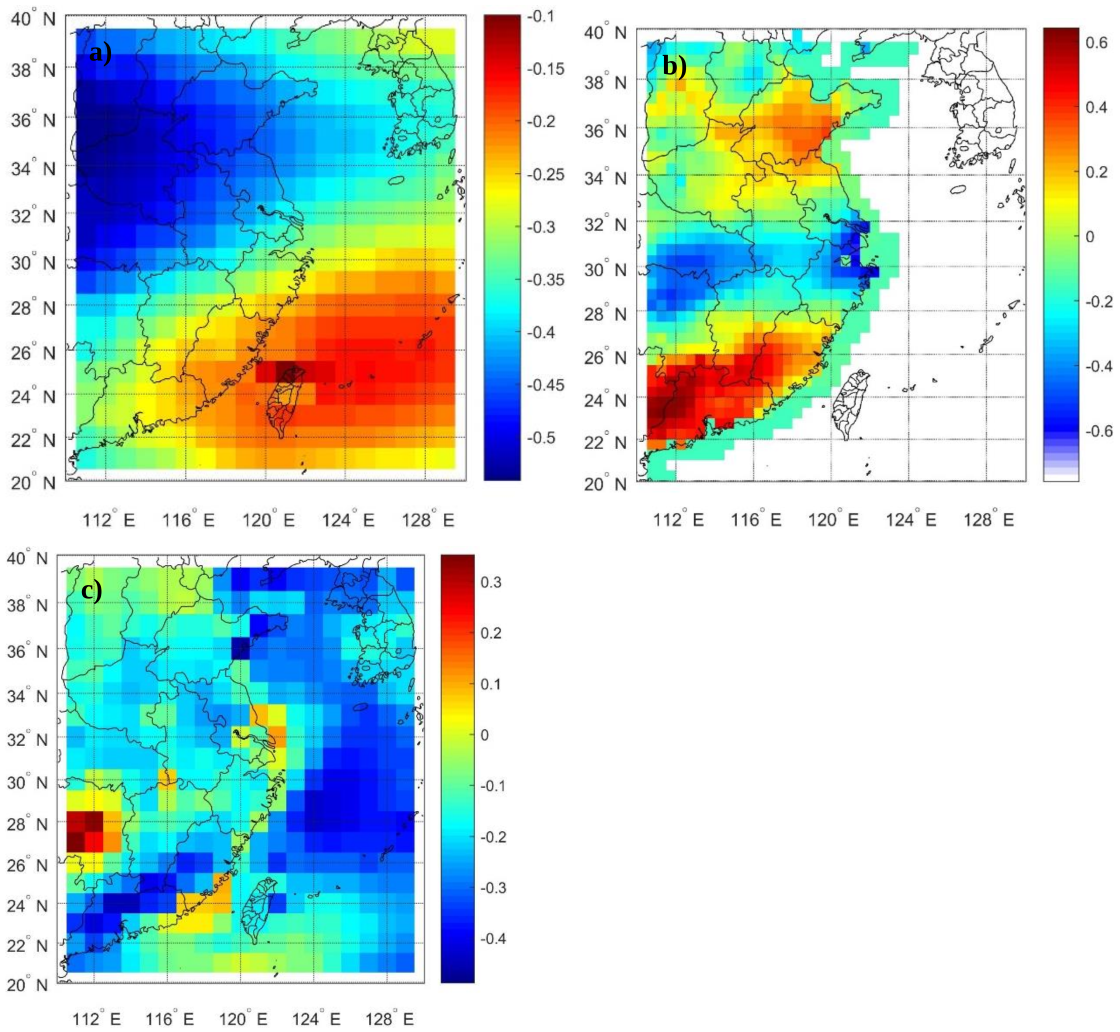

Figure S7. The correlation between PC2 and annual winter mean **a)** 500 hPa geopotential height, **b)** precipitation, and **c)** PBL height in each grid for the period of 2000-2015. All plots were generated using MATLAB, version 9.0.0.341360, https://cn.mathworks.com/support/?s_tid=gn_supp).
